# Paraoxonase 1 (PON1) Q192R genotypes and their interaction with smoking strongly increase atherogenicity and the Framingham risk score

**DOI:** 10.1590/2359-3997000000184

**Published:** 2016-08-23

**Authors:** Andre de Souza-Nogueira, Alissana Ester Camargo, Felipe Assan Remondi, Monica Maria Bastos Paoliello, Rebecca J. Richter, Clement E. Furlong, Decio Sabbatini Barbosa, Michael Maes, Estefania Gastaldello Moreira

**Affiliations:** 1 Universidade Estadual de Londrina Londrina PR Brasil Programa de Pós-Graduação em Ciências da Saúde, Universidade Estadual de Londrina (UEL), Londrina, PR, Brasil; 2 Departamento de Patologia, Análises Clínicas e Toxicológicas UEL Londrina PR Brasil Departamento de Patologia, Análises Clínicas e Toxicológicas, UEL, Londrina, PR, Brasil; 3 Secretaria de Saúde do Estado do Paraná Londrina PR Brasil Secretaria de Saúde do Estado do Paraná, Londrina, PR, Brasil; 4 UEL Londrina PR Brasil Programa de Pós-Graduação em Saúde Pública, UEL, Londrina, PR, Brasil; 5 Departamento de Ciências do Genoma e Medicina Divisão de Genética Médica Universidade de Washington Seattle Washington EUA Departamento de Ciências do Genoma e Medicina, Divisão de Genética Médica, Universidade de Washington, Seattle, Washington, EUA; 6 Centro de Pesquisa de Impacto Estratégico Faculdade de Medicina Universidade de Deakin Geelong Victoria Austrália Centro de Pesquisa de Impacto Estratégico, Faculdade de Medicina, Universidade de Deakin, Geelong, Victoria, Austrália; 7 Departamento de Ciências Fisiológicas UEL Londrina PR Brasil Departamento de Ciências Fisiológicas, UEL, Londrina, PR, Brasil

**Keywords:** Atherogenic index, cardiovascular risk, Framingham score risk, lipid profile, paraoxonase 1, smoking

## Abstract

**Objective:**

Paraoxonase 1 (PON1) polymorphisms are associated with an increased susceptibility to cardiovascular disease. PON1 Q192R polymorphism (rs662) partially determine PON1 hydrolytic activity and protect against oxidation of LDL and HDL. This study aimed to delineate the association of PON1 status (functional 192 genotype and plasma activity levels) and atherogenicity in urbans residents aged 40 years or more.

**Materials and methods:**

Anthropometric data, lipid profiles, the atherogenic index of the plasma (AIP) and Framingham score risk were measured. Three kinetic assays were conducted to assay PON1 status using phenylacetate and 4-(chloromethyl)phenyl acetate as substrates.

**Results:**

Smoking per se did not significantly impact the AIP but the interaction PON1 genotype by smoking significantly increased the AIP. In subjects with the RR genotype smoking increased the AIP index from (estimated mean ± SEM) -0.038 ± 0.039 to 0.224 ± 0.094. The QR genotype increased the Framingham risk index by around 1.3 points. Smoking by RR genotype carriers significantly increased the Framingham risk score (17.23 ± 2.04) as compared to smoking (13.00 ± 1.06) and non-smoking (7.79 ± 0.70) by QQ+QR genotype carriers. The interaction RR genotype by smoking was a more important predictor (odds ratio = 7.90) of an increased Framingham risk score (> 20) than smoking per se (odds ratio = 2.73). The interaction smoking by RR genotype carriers significantly increased triglycerides and lowered HDL cholesterol.

**Conclusion:**

Smoking per se has no (AIP) or a mild (Framingham risk score) effect on atherogenicity, while the interaction smoking by PON1 RR genotype has a clinically highly significant impact on atherogenicity.

## INTRODUCTION

Human serum paraoxonase 1 enzyme (PON1; EC 3.1.8.1) is a glycoprotein synthesized in the liver and mostly bound to high density lipoproteins (HDL) particles in plasma. PON1 protects low density lipoproteins (LDL) (1) and HDL (2) from oxidation possibly by hydrolyzing phospholipid or cholesteryl ester hydroperoxides (3). Evidence for the role of PON1 in the antioxidant property of HDL is provided by the findings that PON1 knockout mice are more susceptible to atherosclerosis when fed on a fat-rich diet (4).

PON1 is polymorphic and single nucleotides polymorphisms (SNP) have been described both in the promoter and coding regions of the *PON1* gene. The most studied polymorphism in the coding region (rs 662) results in an exchange of a glutamine (Q) by an arginine (R) at position 192 of the amino acid sequence. This PON1 Q192R polymorphism modulates the catalytic activity of PON1 but the direction of this change is substrate-dependent (5,6).

Different PON1 genotypes have been investigated as markers of susceptibility to cardiovascular disease (7). The PON1 Q192R polymorphism determines in part PON1 hydrolytic activity and therefore plays a role in the protection of LDL and HDL against oxidation. These effects are, however, heavily debated since both the PON1*192Q allele (8-10) and the PON1*192R allele (11,12) have been associated with increased risk for cardiovascular disease, while there are also studies describing no association (13). One factor that may explain the contradictory results is the approach used to evaluate PON1. Most studies have investigated PON1 genotype and did not include the measurement of PON1 plasmatic activity. Total PON1 plasmatic activity is negatively related to increased risk for cardiovascular disease (8,14-16). Therefore, the measurement of PON1 status, that provides a functional assignment of an individual’s PON1 Q192R polymorphism and their total total PON1 plasma activity (17) is a more reliable marker than genotype or plasma activity measurement alone (7,18).

Dyslipidemia is one of the major risk factors for cardiovascular disorders. Increased levels of triglycerides and total and LDL cholesterol are positively associated with cardiovascular disorders whereas high HDL levels have a protective effect. Different combinations of lipid profile parameters and other risk factors can be used to identify high risk individuals (19). One of these indexes is the atherogenic index of plasma (AIP), which is the relationship between log triglycerides and HDL cholesterol and correlates closely with LDL particle size (20). AIP has been considered a reliable index for atherosclerosis because it is known that the smaller the HDL-c particle, the higher the risk of esterification by lecithin cholesterol acyltransferase and consequently atherosclerosis development (20). Another index is the Framingham risk score, which takes into consideration variables such as age, sex, smoking status, lipid profile and hypertension to estimate a 10-year risk of a cardiovascular disease event (21).

Considering the controversy related to the influence of PON1 Q192R on lipid profile and that no study has examined whether this polymorphism is associated with the AIP and Framingham index, this study aimed to delineate the association of PON1 status with those indexes in randomly selected urban residents aged 40 years or more.

## MATERIALS AND METHODS

### Subjects

This study is part of a main study on cardiovascular health (cross sectional, population based study) conducted by the Public Health Department, State University of Londrina, Brazil. The study participants were urban residents, aged 40 years or more, randomly selected from the city of Cambe, Parana State, Brazil. The study population consisted of 1180 participants (with standardized interviews) and 967 participants (interviews and blood samplings) (22). Serum samples were available from 700 individuals. We excluded subjects who used multiple antiviral (1 individual) or immunossupressant (4 individuals) drugs, fish oil or omega-3 (3 individuals), lithium (4 individuals) and allopurinol (3 individuals). Based on these exclusion criteria a subset of 685 individuals was selected for this study. The study was approved by the local Ethics Committee on Human Research (CAAE: 0192.0.268.000-10). All subjects gave written informed consent to participate in the study.

### Demographic and anthropometric data

Subjects were interviewed before blood sampling in order to collect socio-demographic data (sex, age, smoking and use of medication) and anthropometric variables (weight, height and waist circumference). Blood pressure was verified using the *Omron HEM-742INT* after 10 min of rest while the subject was seated, and the mean of two recordings was used. Waist circumference was measured using a tape with the subject standing at the level of midway between the lower rib margin and the iliac crest. Body mass index (BMI) was calculated dividing the weight in kg by the height in meters squared. Subjects were divided into non smokers (n = 564) and current smokers (n = 121). The latter group comprised 109 current smokers who smoked daily and 12 subjects who smoked regularly but not daily. The mean number of cigarettes/day was 15.2 (±10.2, standard deviation).

### Laboratory measurements

After an overnight fast, blood samples were collected by venipuncture into tubes without anticoagulant. The samples were immediately centrifuged and the serum was aliquoted and stored at -80°C until processing. The lipid profile was assessed by enzymatic colorimetric methods in an automated clinical chemistry system (Dimension RXL, Siemens, USA). LDL cholesterol was calculated using Friedewald formula (23). LDL was not estimated in 9 individuals with triglyceride values higher than 400 mg/dL. The interassay coefficients of variation for all lipid markers were lower than 10%.

**Figure 1:**
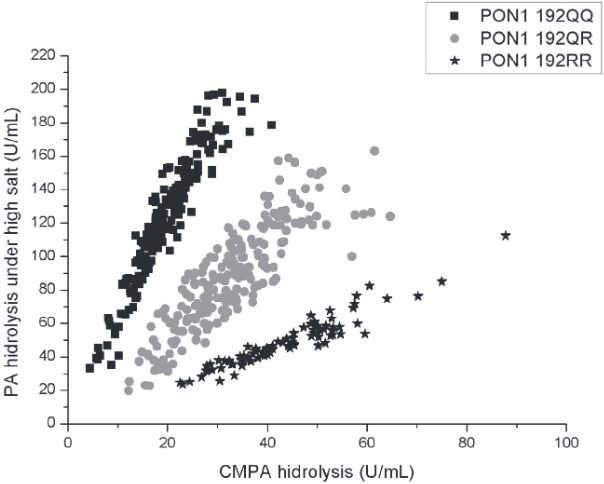
Population distribution plot of hydrolysis of 4-(chloromethyl)phenyl acetate versus phenylacetate. Each data point indicates one individual.

PON1 status was determined through three kinetic assays (24). To stratify individuals in the functional genotypes for the PON1 Q192R polymorphism (PON1_192Q/Q_, PON1_192Q/R_, PON1_192R/R_) the substrates used were phenylacetate (PA, Sigma, USA) and 4-(chloromethyl)phenyl acetate (CMPA, Sigma, USA). Q allozyme presents low efficiency to metabolize CMPA whereas both alloforms hydrolyze PA with approximately the same efficiency. The reaction with PA is conducted under high salt condition in order to partially inhibit the activity of the R allozyme thus providing a better resolution of the three PON1_192_ functional genotypes. The analysis was conducted in a spectrophotometer microplate reader (EnSpire, Perkin Elmer, USA). All assays were carried out in triplicate and replicates that varied by 10% or greater were repeated. Briefly, CMPA hydrolysis was measured at 280 nm for 4 min at 25ºC using 20 µL of plasma diluted 1:40 in dilution buffer [20 mmol/L Tris-HCl (pH 8.0), 1.0 mmol/L CaCl2]. PA hydrolysis under high salt conditions were measured at 270 nm for 4 min at 25ºC using 20 µL of plasma diluted 1:40 in dilution buffer. High salt media was composed by PA added to 2 mol/L NaCl, 20 mmol/L Tris-HCl (pH 8.0), 1.0 mmol/L CaCl2. The results obtained with these two assays were used to plot a 2-dimensional enzyme activity graphic that displays rates of PA hydrolysis under high salt conditions versus CMPA hydrolysis. Figure 1 shows the classification of the subjects from this study. A third assay that measures rates of PA hydrolysis at low salt concentration reveals plasma PON1 activity since under this assay condition, the PON1 Q192R polymorphism does not influence PON1 catalytic activity against PA (25). For this assay, rates of hydrolysis of PA were measured at 270 nm for 4 min at 25ºC using 20 µL of plasma diluted 1:80 in dilution buffer. Only the linear initial rates of substrate hydrolysis were measured for all calculations.

### Indexes of cardiovascular disease risk

AIP was calculated as log triglycerides/HDL cholesterol (20). Framingham score was calculated taking into consideration the algorithms sex, age, concentrations of total cholesterol and HDL, smoking, systolic blood pressure, and self-declared treatment for hypertension status. The obtained Framingham score was then converted into the Framingham absolute risk to have a cardiovascular disease in 10 years (26).

### Statistics

We used analyses of variance to check differences in continuous variables between subjects allocated to different study groups (e.g. AIP groups). If significant, the Tukey test was employed to check planned comparisons among multiple treatment means. Multivariate and univariate general linear model (GLM) analyses were used to examine the effects of explanatory variables, either categories or continuous variables, on the AIP or Framingham risk score. Pairwise comparisons among estimated marginal means were performed using the Bonferroni method. Correlations between variables were calculated using Pearson’s correlation coefficients. Analyses of contingence tables (Chi-square test) were used to ascertain the distribution of variables among study groups. We used binary logistic regression analyses to check the association between an increased AIP and Framingham risk index and explanatory variables including PON1 Q192R genotypes, smoking (and their interactions), sex, abdominal circumference, age, etc. We log-transformed variables when necessary in order to normalize data distribution. The SPSS (Windows version 19) was employed to analyze all data. Statistical significance was set at p = 0.05, two tailed.

## RESULTS

### PON1 status

From the 685 participants, plasma from 484 was available for PON1 status determination. Functional PON1 Q192R genotyping failed to unveil the genotype of 8 participants. According to their PON1 Q192R polymorphism, 476 participants were classified as homozygous for PON1*192Q allele (186 subjects; 39.1%); heterozygous (212 subjects; 44.5%) or homozygous for the PON1*192R allele (78 subjects; 16.4%). This population is in Hardy-Weinberg equilibrium. Regarding PON1 activity, the data in Figure 1 show the large variability of this measure (arylesterase activity in U/mL) in each functional genotype: 46.9 to 377.3 for PON1 192QQ individuals, 45.3 to 348.6 for PON1 192QR individuals and 81.29 to 269.8 for PON1 192RR individuals.

### Socio-demographic and clinical data

Table 1 shows the socio-demographic and clinical data of the subjects in this study. The subjects were divided in three groups, those with normal AIP (< 0.11), medium AIP (0.11 - 0.21), and high AIP (> 0.21). There were no significant differences in age, PON1 Q192R genotypes, PON1 total activity, between the three study groups. There were significant more males in the high AIP group. BMI was significantly higher in subjects with a medium and high AIP as compared to those with a normal AIP. Waist circumference was significantly different among the three groups and increased from the normal, medium to high AIP group. All pairwise comparisons performed on total cholesterol, HDL cholesterol, triglyceride levels and AIP were significantly different between the three groups. The Framingham score was significantly higher in subjects with medium and high AIP values than in those with normal values. The Framingham risk index was significantly higher in subjects with high AIP values than in the two other subgroups. There were significant correlations between the AIP and Framingham score (r = 0.314, p < 0.001, n = 678) and Framingham risk (r = 0.277, p < 0.001, n = 678). The Framingham score and risk were significantly intercorrelated (r = 0.586, p < 0.001, n = 678). In the current smokers, there were no significant associations between the number of cigarettes smoked per day and the Framingham score, Framingham risk index, AIP, lipid levels, PON1 Q192R genotypes and PON1 total activity.

**Table 1:** Socio-demographic and clinical data of subjects with a normal (< 0.11), moderately increased (0.11 – 0.21) and highly increased (> 0.21) atherogenic index of plasma (AIP)

Variable	Normal (AIP < 0.11)	Medium (0.11 < AIP < 0.21)	High (AIP > 0.21)	F or Χ^2^	df	P value
Age (years)	55.0 (10.7)	55.4 (9.8)	54.8 (9.7)	0.11	2/282	0.900
Sex (female/male)	237/155	59/29	92/113*^#^	17.76	2	< 0.001
BMI (kg/m^2^)	26.5 (4.9)	29.2 (5.1)*	30.1 (4.6)*	41.00	2/675	< 0.001
Waist circumference (cm)	90.0 (12.5)	97.6 (11.5)	101.5 (10.5)	56.09	2/680	< 0.001
PON1 functional genotype	–	–	–	5.01	4	0.286
QQ (number of subjects)	110	26	50	–	–	–
QR (number of subjects)	112	33	67			
RR (number of subjects)	52	10	16			
PON1 total activity (U/mL)^a^	177.5 (60.8)	185.0 (69.2)	186.2 (57.0)	1.11	2/481	0.330
Total cholesterol (mg/dL)	195.9 (35.3)	209.9 (34.2)*	222.3 (42.7)*^#^	34.02	2/682	< 0.001
HDL cholesterol (mg/dL)	53.6 (14.6)	43.8 (7.9)*	38.8 (9.1)*^#^	119.58	2/682	< 0.001
Triglycerides (mg/dL)	85.6 (28.3)	144.1 (26.8)*	281.3 (239.6)*^#^	592.87	2/682	< 0.001
AIP	-0.168 (0.198)	0.157 (0.029)*	0.450 (0.227)*^#^	689.62	2/682	< 0.001
Framingham score	11.62 (4.94)	13.61 (4.57)*	14.14 (4.50)*	20.67	2/675	< 0.001
Framingham risk	5.90 (6.72)	7.41 (7.87)	10.28 (8.18)*^#^	23.64	2/675	< 0.001

Data are shown as mean (standard deviation). Continuous variables were analyzed by ANOVA complemented with Tukey test whereas discrete variables were analyzed by Chi-square test.

* p < 0.05 compared to Normal AIP group; ^#^ p < 0.05 compared to Medium AIP group; ^a^ PON1 total activity reflects AREase activity determined under low salt condition. BMI: body mass index; HDL: high density lipoprotein; AIP: atherogenic index of the plasma.

### Logistic regression of odds of AIP > 0.21 versus lower AIP

Table 2 shows the results of a logistic regression analysis with individuals with an increased AIP (that is > 0.21) versus those with a normal or medium index as reference group. Automatic stepwise binary regression analysis showed that subjects with high AIP values were significantly distinguished (c^2^ = 66.23, df = 3, p < 0.001; Nagelkerke = 0.188) from those with a normal/medium index. Waist circumference and the interaction term RR genotype x smoking were positively associated with the high AIP group, whereas female sex was negatively associated. The significant interaction pattern between PON1 RR genotype by smoking shows that smoking by RR carriers increases the odds to belong to the high AIP group. Entering smoking (Wald = 0.34, df = 1, p = 0.558) separately showed that smoking alone was not associated with a high AIP and that the effects of the interaction RR genotype x smoking remained significant (Wald = 6.18, df = 1, p = 0.013).

**Table 2:** Results of binary logistic regression analysis with subjects with increased atherogenic index of plasma (AIP ≥ 0.21) as dependent variable and subjects with AIP < 0.210 as reference group

Significant explanatory variables	Wald	Df	p value	Odds Ratio	95% CI
Waist circumference	43.10	1	< 0.001	1.07	1.05 – 1.09
Female sex	11.62	1	0.001	0.47	0.30 – 0.73
RR genotype x current smoking	6.13	1	0.013	4.86	1.39 – 16.99

95% CI: 95% confidence intervals (lower – upper).

### AIP and HDL cholesterol and triglyceride levels

Table 3 shows the results of multivariate GLM analysis with AIP and HDL cholesterol and triglycerides levels as dependent variables and waist circumference, sex, age, plasma PON1 activity, smoking and the interaction term RR x smoking as explanatory variables (entered as factors or covariates). Multivariate tests showed that sex, age, waist circumference, total PON1 activity and the interaction RR x smoking were significantly associated with AIP, HDL cholesterol and triglycerides. Tests of between subjects’ effects showed that sex was significantly associated with AIP (higher in men); waist circumference positively with AIP and triglycerides, but negatively with HDL cholesterol; and the interaction RR x smoking positively with AIP and triglycerides, but negatively with HDL cholesterol. There was also a significant inverse correlation between age and HDL cholesterol and a positive between total PON1 activity and triglycerides. We found that 20.1% of the variance in AIP was explained (F = 16.67, df = 7/464, p < 0.001) by the regression on sex, waist circumference and the interaction between smoking and RR genotype. In subjects with the RR genotype, smoking increased the AIP index from -0.038 ± 0.039 to 0.224 ± 0.094 (estimated marginal means ± standard error). Seventeen point three percent of the variance in triglycerides (F = 13.88, df = 1, p < 0.001) was explained by the regression on waist circumference, total PON1 activity and the interaction smoking x RR genotype. Thirteen point six percent of the variance in HDL cholesterol was explained by the regression on age, waist circumference and the interaction smoking x RR genotype.

**Table 3:** Results of multivariate general linear model (GLM) analyses with atherogenic index of plasma (AIP), high density lipoprotein cholesterol (HDL) and triglycerides (TG) as dependent variables

Analyses	Dependent variable(s)	Explanatory variable	F	df	P value
Multivariate	AIP, HDL and TG	Sex	4.44	2/463	0.012
		Smoking	1.86	2/463	0.157
		Age	6.35	2/463	0.002
		Waist circumference	47.66	2/463	< 0.001
		PON1 activity	5.94	2/463	0.003
		RR x current smoking	3.61	2/928	0.006
Significant Univariate	AIP	Sex	4.87	1/464	0.028
		Waist circumference	93.22	1/464	< 0.001
		RR x current smoking	5.63	1/464	0.004
	HDL	Age	10.99	1/464	0.001
		Waist circumference	55.74	1/464	< 0.001
		RR x current smoking	5.54	1/464	0.004
	TG	Waist circumference	75.05	1/464	< 0.001
		PON1 activity	5.22	1/464	0.023
		RR x current smoking	3.89	1/464	0.021

We have also examined the possible intervening effects of the use of different medications on the AIP index. Table 4 shows the effects of the different drugs used by the participants on the AIP. Subjects using oral hypoglycemics, oral hypoglycemics and/or insulin, hypolipidemics (fibrates and/or statins) and antihypertensives showed higher AIP values than subjects who did not take these medications. Forced entry of those 5 different medication variables in the multivariate GLM analysis displayed in Table 3 shows that after considering the effects of these 5 drugs, age (F = 7.28, df = 2/458, p = 0.001), sex (F = 4.86, df = 2/458, p = 0.008), waist circumference (F = 33.90, df = 2/458, p < 0.001), PON1 activity (F = 5.59, df = 2/458, p = 0.004) and the smoking x RR interaction (F = 3.29, df = 4/918, p = 0.011) remained significant. Only use of antihypertensives (F = 4.50, df = 2/258, p = 0.012) was significant in this multivariate GLM analysis, whereas the other 4 drugs were not significant.

**Table 4:** Differences in the atherogenic index of plasma (AIP) between subjects with and without medications

Drug	No use	Use	F	Df	P
Oral hypoglycemics	0.044 (0.336) n = 626	0.211 (0.330) n = 59	13.37	1/683	< 0.001
Oral hypoglycemics and/or insulin	0.045 (0.336) n = 622	0.195 (0.334) n = 63	11.53	1/683	0.001
Antihypertensives	0.006 (0.330) n = 430	0.147 (0.320) n = 255	28.75	1/683	< 0.001
Statins	0.053 (0.348) n = 614	0.109 (0.242) n = 71	1.78	1/683	0.182
Fibrates	0.053 (0.338) n = 672	0.324 (0.265) n = 13	8.21	1/683	0.004
Statins and/or fibrates	0.049 (0.347) n = 605	0.132 (0.254) n = 80	4.26	1/683	0.039
Dipyrone	0.050 (0.333) n = 528	0.086 (0.365) n = 157	1.31	1/683	0.252
Aspirin	0.050 (0.335) n = 614	0.131 (0.359) n = 71	6.63	1/683	0.057
Any non-steroidal antiinflamatory	0.040 (0.334) n = 372	0.080 (0.343) n = 313	2.42	1/683	0.120

All results are shown as mean (standard deviation). F: results of analyses of variance with AIP as dependent variable and the different drugs as categories (use versus no use).

### Framingham risk index

Table 5 shows that a higher waist circumference, QR genotype and groups according to RR and smoking were significantly associated with the Framingham risk index after considering the effects of the significant drug variables, i.e., use of hypoglycemics, dipyrone and aspirin. The same table also shows the estimated marginal means in the genotypic groups, i.e. QR genotype and the groups divided according to smoking and the RR genotype. Thus, The QR genotype increases the Framingham risk index by around 1.3 points, whereas smoking by RR genotype carriers increases the index by 4.2 points versus smoking QQ+QR genotype carriers and 9.44 points when compared to non-smoking QQ+QR carriers. The use of dipyrone lowered the index from 13.56 (±0.95) to 11.79 (±1.09), whereas aspirin increased the index from 11.26 (±0.94) to 14.09 (±1.23). The use of hypoglycemics was associated with a higher Framingham risk index (11.29 ± 0.86 versus 14.05 ± 1.34).

**Table 5:** Univariate general linear model (GLM) analysis with Framingham index as dependent variable

Explanatory variables	F	Df	p value	Partial ε^2^
Model	15.28	7/461	< 0.001	188
Waist circumference	23.61	1/461	< 0.001	49
QR genotype	4.35	1/461	0.038	0.9
RR x current smoking (3 groups)	26.45	2/461	< 0.001	10.3
Hypoglycemics	5.24	1/461	0.023	1.1
Dipyrone Aspirin	5.99 7.20	1/461 1/461	0.015 0.008	1.3 1.5

**Estimated marginal means**	**Mean (SE)**			

Not QR^&^	12.01 (0.98)			
QR^&^	13.33 (1.03)			
--------------------------	------------------	------------------		
Not RR + not smoking^$^	7.79 (0.70)			
Not RR + current smoking^$^	13.00 (1.06)*			
RR + current smoking^$^	17.23 (2.04)*^#^			

^&^ Estimated marginal mean (standard error) values in QR carriers versus QQ and RR carriers.

^$^ Estimated marginal mean (standard error) values in smoking RR carriers versus smoking or non-smoking QQ + QR carriers.

* P < 0.05 compared to not RR + not smoking.

^#^ P < 0.05 compared to not RR + current smoking.

Table 6 shows the results of a logistic regression analysis with the group of subjects with a high Framingham index (> 20) as dependent variable and the other subjects as reference group. Waist circumference, the QR genotype, use of hypoglycemics and groups divided according to smoking and the RR genotype were significantly associated with a higher Framingham risk index (c^2^ = 35.44, df = 6, p < 0.001; Nagelkerke = 0.147), whereas dipyrone was inversely associated (c^2^ = 35.44, df = 6, p < 0.001; Nagelkerke = 0.147). The RR genotype in combination with smoking resulted in a much higher odds ratio (i.e. 7.90) than smoking alone (i.e. 2.73).

**Table 6:** Results of binary logistic regression analysis with Framingham index > 20 as dependent variable and subjects with Framingham index < 20 as reference group

Significant explanatory variables	Wald	df	p value	Odds Ratio	95% CI
Waist circumference	6.82	1	0.009	1.03	1.01 – 1.06
QR genotype	5.26	1	0.022	2.79	1.16 – 6.71
Current smoking x QQ +QR genotype carriers	6.51	1	0.011	2.73	1.26 – 5.89
Current smoking x RR genotype carriers	7.53	1	0.006	7.90	1.81 – 34.60
Hypoglycemics	5.26	1	0.022	2.79	1.11 – 6.71
Dipyrone	4.97	1	0.026	0.33	0.13 – 0.88

95% CI: 95% confidence intervals (lower – upper).

## DISCUSSION

To the best of our knowledge, this is the first study examining the association between PON1-Q192R polymorphism and atherogenic indexes, i.e. AIP and Framingham score risk, in a general population. The major finding of this study is that the PON1 Q192R polymorphism is associated with increased atherogenicity, i.e. the PON1-QR genotype increases the Framingham risk score and the interaction between smoking by PON1-RR carriers is associated with an increased AIP and Framingham score risk and by lowered HDL-cholesterol and increased triglyceride levels.

An atherogenic lipid profile and increased incidence of cardiovascular disease have been described in individuals homozygous to the PON1*192R allele (12,27,28). In a study performed on non-cardiovascular patients, PON1-RR smokers had a more atherogenic lipid profile (29). The latter could be attributed to a lower hydrolytic activity of the PON1 R192 allozyme towards lipid peroxides as compared to the Q192 isoform (6). On the other hand, smoking induces oxidative stress and is a known risk factor for dyslipidemias. Therefore, the pro-atherogenic state observed in PON1-RR smokers may reflect a synergism between an increased oxidative stress status induced by smoking and a decreased ability to hydrolyze lipid peroxides, assigned by the R192 allozyme. Moreover, the decreased HDL cholesterol could also have resulted from a decrease in its synthesis since the enzyme lecithin-cholesterol acyltransferase can be inactivated in the presence of higher levels of lipid hydroperoxides (30).

It is noteworthy that even though smoking has been reported to be associated with an elevated AIP (31,32), in the present study AIP was not affected by smoking per se but rather by the interaction between smoking and PON1 192QR genotypes. Moreover, the impact of smoking on the Framingham risk index was much higher in PON1-RR individuals as compared to non-smoking PON1-QQ/QR individuals. Atherogenic indexes are considered to be better predictors of increased risk to cardiovascular diseases than each of the lipids (HDL-cholesterol triglycerides, total cholesterol) separately (19,20,33). The AIP for example is considered an indicator of atherogenic dyslipidemia, i.e., the combined occurrence of high fasting blood concentrations of triglycerides and low levels of HDL particles. We found that PON1-RR smokers presented a clinically highly relevant increase of 9.4 points on the Framingham risk index as compared to non-smoking PON1-QQ/QR individuals and thus almost a 10% higher risk for having a myocardial infarction in 10 years.

In the present study there was a positive correlation between PON1 plasmatic activity (arylesterase activity) and triglycerides but not with HDL. Regarding HDL cholesterol, a positive relationship was expected since the majority of PON1 proteins are bound to these particles. Roest and cols. (34) described a weak association between PON1 activity (determined using PA) and HDL. These authors offered two possible explanations for this lack of/weak association: a) PON1 may be associated only with specific subspecies of HDL; b) the low saturation rate of HDL with PON1 (there is a sevenfold excess of HDL particles to bind only one PON1 molecule) which would explain the limited impact of fluctuations in HDL on PON1 concentration in blood. The association between PON1 and lipid metabolism is rather complex and the mechanisms underlying this association remain to be elucidated. At the same time that PON1, due to its protective effect on HDL and LDL, influences lipid metabolism and serum lipoproteins, lipid metabolism can also influence PON1 activity modulating its expression (35,36). Interestingly, van Himbergen and cols. (37) reported a remarkably different association between PON1 arylesterase activity and lipid profile when compared familial combined hyperlipidemia patients with their unaffected relatives. In the relatives, PON1 associated with higher levels of apoliprotein B (apoB), HDL- and LDL-cholesterol. In the patients, conversely, it associated with higher levels of VLDL-cholesterol and triglycerides. Our results indicate that a positive association between PON1 arylesterase and triglycerides may not be restricted to familial combined hyperlipidemia patients. Moreover, our results reinforce the concept that PON1 status determination is a better approach to investigate PON1 influence on lipid profile once activity reached significance as an explanatory variable to triglycerides levels whereas the interaction between PON1 Q192R polymorphism and smoking was one of the explanatory variables to AIP, Framingham score risk, triglycerides and HDL. Having both datasets in multivariate models was a step forward for meaningful interpretation of the results. As expected, male gender significantly predicted increased AIP values whereas waist circumference predicted increased AIP and triglycerides and decreased HDL. These findings reflect the higher levels of HDL in women and the negative impact central obesity has on lipid profile.

Another finding of the present study is that subjects who took drugs to treat cardiometabolic diseases presented higher AIP values. Even though statistical significance was observed for oral hypoglycemics, fibrates and antihypertensives, a similar trend could also be observed for statins and aspirin (Table 4). This finding may be reflecting the influence of cardiometabolic diseases on AIP and suggests that the use of medication does not necessarily bring AIP values to normal levels. For example, even though treatment with fibrates (20) and statins (38) decreases AIP when comparing pre- and post-treatment values, control values are not reached. Moreover, it has been described that in more than 70% of the patients atherogenic risk persists despite of the treatment with hypolipidemics (39). Our data also show a positive association between the Framingham risk index and use of aspirin and hypoglycemic, but a negative association with dipyrone. Moreover, aspirin has cardioprotective activities (40-42). Our study, however, was not designed to examine the effects of drugs on the atherogenic indexes and data interpretation is complex since some subjects presented more than one cardiometabolic disease and various subjects were polymedicated. Most importantly is that after adjusting our data for drug use, AIP remained positively and significantly associated with PON1*192R allele carrier smokers.

Finally, supporting the high influence of European colonization in the south of Brazil, the present study as well as a study published in 2002 (43) describe a higher frequency of PON1192Q/Q and heterozygous than PON1192R/R among the subjects. Population-based studies and meta-analysis have shown that the PON1*192R allele is more common in African population, whereas Q isoform is more frequent in Caucasians (6).

This study has strengths and limitations that must be considered for the interpretation of the results. Firstly, considering the factors that have been described to influence PON1 activity (for a review, see [44]) limitations of our study include lack of information on nutrition and alcohol consumption. Secondly, this is a cross-sectional study and therefore we cannot assert causality. Strengths are that our results were adjusted for many potential confounders, including age, sex, waist circumference, smoking and use of drugs. Most importantly, we included the functional measure of PON1 total plasmatic activity for each subject within each PON1 Q192R phenotypic group. The large variability observed among individuals represent important differences in the individual’s rates of detoxification of endogenous toxic metabolites as well as xenobiotics.

This study shows an important interaction between PON1 192RR functional genotype and smoking. Smoking by PON1 RR carriers was associated by significantly increased AIP, triglyceride levels and a Framingham score risk and by significantly lower levels of HDL cholesterol. The findings provide an example of gene-environment interactions that increase cardiovascular risk.
